# Enriched Versus Standard NICU Environments and Preterm Brain Development: A Systematic Review of Structural, Diffusion, and Functional Neuroimaging Studies

**DOI:** 10.7759/cureus.114433

**Published:** 2026-08-12

**Authors:** Saritha Tammali, Saritha Kamsetti, Ashok Kumar Urakura, Rakesh Kotha

**Affiliations:** 1 Pediatrics, Niloufer Hospital, Hyderabad, IND; 2 Pediatrics, Government Medical College Vikarabad, Vikarabad, IND; 3 Neonatology, Osmania Medical College, Hyderabad, IND

**Keywords:** brain development, diffusion tensor imaging (dti), neonatal intensive care unit, preterm infant, systematic review

## Abstract

Preterm infants are at increased risk of developing abnormal brain structure. Research has also indicated that the environment of the NICU could affect the early development of the infant's nervous system. Methods used to evaluate brain development and white matter maturation include structural magnetic resonance imaging (MRI), diffusion tensor imaging, and selected functional MRI techniques. This qualitative systematic review without meta-analysis synthesized evidence from neuroimaging studies evaluating enriched versus standard NICU environments in preterm infants. A systematic literature search of PubMed and Scopus databases identified randomized controlled trials (RCTs) and prospective studies reporting neuroimaging outcomes. For RCTs, the risk of bias was assessed using the Cochrane Risk of Bias tool, and cohort studies were evaluated using the Newcastle-Ottawa scale. A quantitative meta-analysis was considered but was not feasible because of substantial heterogeneity in interventions, imaging modalities, outcome measures, regions of interest, follow-up duration, and reporting standards. Therefore, findings were synthesized qualitatively. Each included study evaluated one or more enriched NICU interventions, including Newborn Individualized Developmental Care and Assessment Program (NIDCAP)-based developmental care, single-family-room care, structured music interventions, parent-sensitivity interventions, and stress-reduction strategies. Several included studies reported that NIDCAP-based developmental care and structured music interventions were associated with favorable neurodevelopmental or neuroimaging findings, whereas evidence for single-family-room care was mixed. Separate observational studies found that greater procedural pain and NICU stress exposure were associated with less favorable neuroimaging outcomes. However, heterogeneity, small sample sizes, follow-up publications from some parent cohorts, and residual confounding limit causal inference and preclude firm clinical recommendations.

## Introduction and background

Preterm birth is defined as birth before 37 completed weeks of gestation and interrupts a critical period of rapid brain growth and maturation. It is associated with alterations in white matter development, subcortical growth, cortical organization, and large-scale neural connectivity, which contribute to an increased risk of adverse neurodevelopmental outcomes [1]. The neonatal intensive care unit (NICU) environment also plays an important role in shaping early brain development. Environmental factors such as sensory stimulation, infant handling, parental involvement, pain exposure, and stress may influence structural and functional brain maturation during this vulnerable developmental period [1].

Advances in neonatal neuroimaging have enabled objective assessment of early brain development using structural magnetic resonance imaging (MRI), diffusion tensor imaging (DTI), and, in selected studies, functional MRI (fMRI). Structural MRI evaluates brain morphology and regional brain volumes, whereas DTI provides quantitative measures of white matter microstructure and maturation. fMRI complements these techniques by assessing functional brain connectivity and network organization, thereby providing additional insights into the effects of early environmental exposures on the developing preterm brain [2].

Experimental and observational studies suggest that enriched developmental care interventions, including the Newborn Individualized Developmental Care and Assessment Program (NIDCAP), structured music interventions, parent-sensitive developmental care, and other neuroprotective strategies, may support brain maturation by reducing environmental stress and promoting appropriate sensory experiences. Conversely, repeated procedural pain, prolonged NICU stress, and inconsistent caregiving have been associated with alterations in white matter development and functional connectivity [3]. However, much of the available evidence remains heterogeneous with respect to study design, intervention type, neuroimaging modality, timing of assessment, and outcome measures.

Earlier reviews frequently combined intervention studies and observational exposure studies without clearly distinguishing their methodological differences, thereby limiting interpretation of the available evidence. The present systematic review without meta-analysis addresses this limitation by categorizing enriched NICU interventions separately from observational stress- and pain-exposure studies while applying a qualitative evidence-synthesis approach. This framework enables interpretation of intervention-specific findings without implying direct comparative effects between different study designs or exposure categories.

Because substantial heterogeneity exists across interventions, neuroimaging protocols, regions of interest, follow-up duration, and reported outcomes, quantitative pooling of results was considered inappropriate. Therefore, this review adopts a qualitative systematic review design and emphasizes cautious interpretation of the available evidence rather than causal inference or comparative effectiveness.

The aim of this systematic review without meta-analysis is to synthesize and critically evaluate evidence from structural MRI, diffusion MRI, and selected fMRI studies examining enriched versus standard NICU environments in preterm infants. By applying a structured qualitative synthesis and comprehensive risk-of-bias assessment, this review identifies intervention-specific neuroimaging findings, summarizes methodological strengths and limitations of the available literature, and highlights evidence gaps that currently preclude quantitative meta-analysis and firm clinical recommendations.

## Review

Methods

Protocol and Reporting

**Figure 1:**
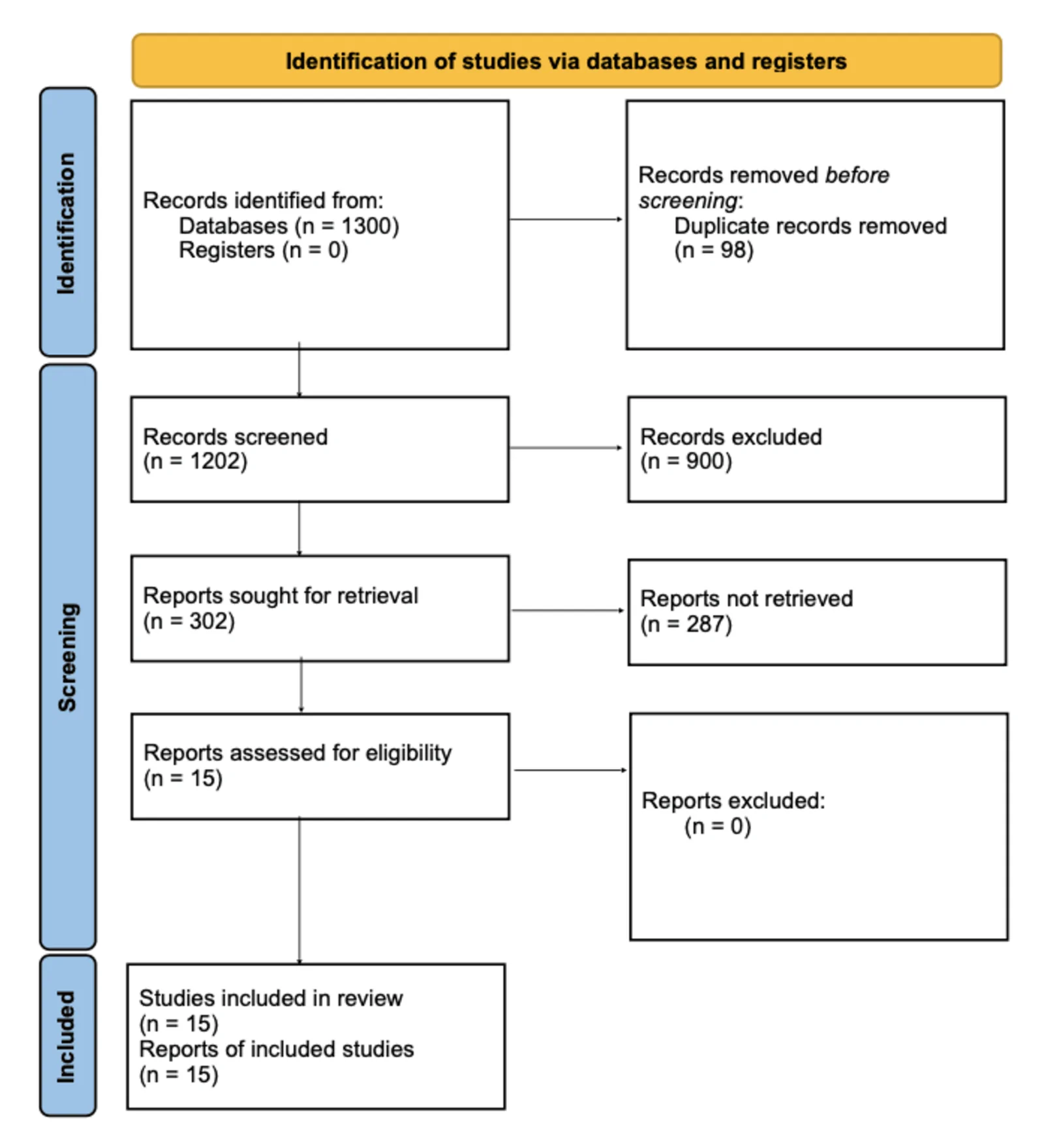
PRISMA 2020 flow diagram of study selection PRISMA: Preferred Reporting Items for Systematic Reviews and Meta-Analyses

This review followed the Preferred Reporting Items for Systematic Reviews and Meta-Analyses (PRISMA) 2020 guidelines [4] and was conducted as a qualitative systematic review without meta-analysis. Study identification, screening, retrieval, eligibility assessment, and inclusion were performed according to PRISMA 2020 recommendations. The PRISMA flow diagram (Figure 1) was revised using standard PRISMA terminology to distinguish records identified, reports sought for retrieval, reports not retrieved, reports assessed for eligibility, and reports included in the qualitative synthesis. This review was conducted collaboratively by Niloufer Hospital/Osmania Medical College, Hyderabad, India, and Government Medical College Vikarabad.

The database search identified 1,300 records, of which 98 duplicate records were removed before screening. The titles and abstracts of 1,202 records were screened, and 900 records were excluded. A total of 302 reports were sought for retrieval. Of these, 287 reports could not be retrieved because they were conference abstracts, inaccessible publications, non-English articles, or archived reports unavailable for full-text review. The remaining 15 reports were assessed for eligibility, and all met the predefined inclusion criteria and were included in the final qualitative synthesis [5-19].

Search Strategy

A comprehensive literature search was performed in PubMed and Scopus on 1 October 2025. The complete Boolean search strategies are presented in Table 1. Search results were exported into a reference-management program, duplicate records were removed, and the remaining records were screened independently by two reviewers. Reference lists of all eligible reports were manually searched to identify additional relevant studies.

**Table 1:** Search strategy used for the literature search NIDCAP, Newborn Individualized Developmental Care and Assessment Program; MRI, magnetic resonance imaging; DTI, diffusion tensor imaging. Source: Developed by the authors.

Database	Search strategy	Filters
PubMed	("preterm"[Title/Abstract] OR "premature"[Title/Abstract] OR "preterm infant"[Title/Abstract] OR "preterm neonate"[Title/Abstract]) AND ("enriched environment"[Title/Abstract] OR "developmental care"[Title/Abstract] OR "NIDCAP"[Title/Abstract] OR "single-family room"[Title/Abstract] OR "sensory intervention"[Title/Abstract] OR "music therapy"[Title/Abstract] OR "kangaroo care"[Title/Abstract] OR "neuroprotective bundle"[Title/Abstract] OR "low-stress protocol"[Title/Abstract] OR "stress"[Title/Abstract] OR "NICU stress"[Title/Abstract] OR "procedural pain"[Title/Abstract] OR "pain exposure"[Title/Abstract] OR "painful procedures"[Title/Abstract]) AND ("MRI"[Title/Abstract] OR "DTI"[Title/Abstract] OR "diffusion tensor imaging"[Title/Abstract] OR "fractional anisotropy"[Title/Abstract] OR "mean diffusivity"[Title/Abstract] OR "brain volume"[Title/Abstract] OR "functional MRI"[Title/Abstract] OR "functional connectivity"[Title/Abstract])	Humans; English language; Publication years 2000–2025
Scopus	TITLE-ABS-KEY(preterm OR premature OR "preterm infant" OR "preterm neonate") AND TITLE-ABS-KEY("enriched environment" OR "developmental care" OR NIDCAP OR "single-family room" OR "sensory intervention" OR "music therapy" OR "kangaroo care" OR "neuroprotective bundle" OR "low-stress protocol" OR stress OR "NICU stress" OR "procedural pain" OR "pain exposure" OR "painful procedures") AND TITLE-ABS-KEY(neuroimaging OR MRI OR DTI OR "diffusion tensor imaging" OR "fractional anisotropy" OR "mean diffusivity" OR "brain volume" OR "functional MRI" OR "functional connectivity")	English language; Publication years 2000–2025

To improve retrieval of observational stress- and pain-related studies, the search strategy included terms related to NICU stress, stress exposure, procedural pain, painful procedures, pain exposure, neuroimaging, magnetic resonance imaging (MRI), diffusion tensor imaging (DTI), diffusion MRI, functional MRI, fractional anisotropy, mean diffusivity, and brain volume, together with enriched developmental care interventions including NIDCAP, single-family-room care, music therapy, kangaroo care, and neuroprotective care bundles.

Eligibility Criteria

Studies were eligible if they included preterm infants born before 37 completed weeks of gestation. Gestational-age categories were defined as extremely preterm (<28 weeks), very preterm (28 to <32 weeks), and moderate-to-late preterm (32 to <37 weeks). Eligible interventions included NIDCAP, individualized developmental care, single-family-room NICU design, structured sensory interventions (including music therapy), parent-sensitive developmental care, neuroprotective care bundles, and observational studies evaluating NICU stress or procedural pain exposure.

Eligible outcomes included structural MRI, diffusion MRI/DTI, and selected fMRI studies evaluating brain development or connectivity at term-equivalent age or later follow-up. Randomized controlled trials, controlled clinical trials, prospective cohort studies, prospective controlled MRI studies, and prospective case-control studies were eligible. Studies lacking neuroimaging outcomes, comparator groups, or involving non-preterm infants were excluded. Definitions of intervention categories are summarized in Table 2.

**Table 2:** Definitions of NICU intervention categories NICU: Neonatal Intensive Care Unit, NIDCAP: Newborn Individualized Developmental Care and Assessment Program Source: Prepared by the authors based on the included studies [5-19].

Category	Definition
NIDCAP/Developmental Care	Standardized, individualized developmental care protocols focusing on infant cues, minimal stress handling, optimized sleep, and structured parental engagement. Includes NIDCAP-guided caregiving plans and training.
Single-Family Room (SFR) NICU	NICU architectural design in which each preterm infant is cared for in a private room allowing continuous parental presence, reduced ambient noise/light, and individualized caregiving.
Sensory Interventions	Structured auditory or multisensory interventions such as music therapy, recorded maternal voice, or regulated sensory input designed to optimize early sensory experience.
Neuroprotective/Stress-Reduction Bundles	Care bundles targeting reduced cumulative stress exposure, improved pain management, and enhanced regulation (e.g., reduced noxious stimuli, clustered care).
Standard NICU (Comparator)	Traditional open-bay NICU layout with routine care practices and variable parental access; baseline comparator across studies.

Risk-of-Bias Assessment

Risk-of-bias assessment was performed independently by two reviewers. Any disagreements were resolved through discussion until consensus was reached.

Randomized controlled trials and randomized follow-up reports were assessed using the Cochrane Risk of Bias 2 (RoB2) tool [20,21], which evaluates bias arising from the randomization process, deviations from intended interventions, missing outcome data, outcome measurement, and selective reporting.

Non-randomized studies were assessed using the Newcastle-Ottawa Scale (NOS) [22], which evaluates selection, comparability, and outcome domains according to study design. Overall risk-of-bias judgments are summarized in Tables 3, 4.

**Table 3:** Risk of bias (RoB2) — randomized controlled trials (six studies)

Study	Randomization process	Deviations from intended interventions	Missing outcome data	Measurement of outcomes	Selective reporting	Overall risk
Als et al., 2004 [5]	Low	Low	Low	Low	Low	Low
Als et al., 2012 [6]	Some concerns	Low	Low	Low	Low	Some concerns
McAnulty et al., 2012 [7]	Low	Low	Low	Low	Low	Low
McAnulty et al., 2013 [8]	Low	Low	Low	Low	Low	Low
Milgrom et al., 2010 [9]	Some concerns	Low	Low	Low	Low	Some concerns
de Almeida et al., 2023 [14]	Some concerns	Low	Low	Low	Low	Some concerns

**Table 4:** Newcastle–Ottawa scale (NOS) — cohort studies (nine studies)

Study (Year) [Ref.]	Selection (0-4)	Comparability (0-2)	Outcome (0-3)	Total (0-9)
Pineda et al., 2014 [10]	4	2	3	9
Lester et al., 2014 [11]	4	2	3	9
Lordier et al., 2019 [12]	4	2	3	9
de Almeida et al., 2020 [13]	4	2	3	9
Smith et al., 2011 [15]	4	1	3	8
Brummelte et al., 2012 [16]	4	1	3	8
Ball et al., 2013 [17]	4	2	3	9
Tortora et al., 2019 [18]	4	1	3	8
Thompson et al., 2014 [19]	4	2	3	9

Data Synthesis and Meta-Analysis Feasibility

A quantitative meta-analysis was considered during study planning. However, calculation of pooled effect estimates was not feasible because of substantial methodological and clinical heterogeneity among the included reports.

The included studies differed considerably with respect to intervention type, imaging modality, scanner field strength, diffusion acquisition protocols, functional imaging techniques, regions of interest, neuroimaging timing, follow-up duration, and reported outcome measures. Several studies also represented follow-up analyses of previously published parent cohorts, precluding independent quantitative pooling.

Accordingly, findings were synthesized using a structured qualitative approach, with results interpreted according to intervention category and neuroimaging modality. Risk-of-bias assessments are summarized in Tables 3, 4.

Results

A total of 15 reports representing several independent study cohorts were included in the qualitative synthesis. Some reports represented follow-up analyses of previously published parent cohorts, and several contributed findings to more than one thematic category. To avoid overinterpretation, findings from follow-up publications were considered complementary rather than independent evidence. The characteristics of the included reports are summarized in Table 5.

**Table 5:** Characteristics of all included studies (N = 15). Several reports represent follow-up analyses of the same parent cohort and should not be interpreted as independent study populations

Study (Year) [Ref.]	Study design	Sample size (n)	Gestational age at birth	Exposure/intervention
Als et al., 2004 [5]	RCT	30	28-33	NIDCAP
Als et al., 2012 [6]	RCT	30 (17 control; 13 intervention)	27-33	NIDCAP (IUGR)
McAnulty et al., 2012 [7]	RCT follow-up	30 original; 23 follow-up	29-33	NIDCAP (low-risk)
McAnulty et al., 2013 [8]	RCT follow-up	30 original; 23 follow-up	27-33	NIDCAP (IUGR)
Milgrom et al., 2010 [9]	RCT	45	<30	Parental sensitivity training
Pineda et al., 2014 [10]	Prospective cohort	136	<30	Private room vs open ward
Lester et al., 2014 [11]	Prospective quasi-experimental cohort	403 (151 open bay; 252 SFR)	<1500 g birth weight	Single-family room
Lordier et al., 2019 [12]	Controlled clinical trial	39 preterm	Preterm	Music intervention
de Almeida et al., 2020 [13]	Prospective controlled MRI study	30 preterm + 15 term	<32	Music intervention
Sa de Almeida et al., 2023 [14]	Blinded RCT	54 recruited; 40 analyzed	<32	Music intervention
Smith et al., 2011 [15]	Prospective cohort	NR	<30	NICU stress exposure
Brummelte et al., 2012 [16]	Prospective cohort	86	24-32	Procedural pain exposure
Ball et al., 2013 [17]	Prospective cohort	47 preterm + 18 term	Median 28+3 (preterm)	Prematurity comparison
Tortora et al., 2019 [18]	Prospective case-control	46 (33 exposed; 13 controls)	Mean 27.9 / 31.2	Pain exposure and fMRI
Thompson et al., 2014 [19]	Prospective cohort	Approx. 96 very preterm + 20 term	<30 or <1250 g	DTI and 7-year outcomes

NIDCAP and Developmental-Care Studies (Five Reports)

Als et al. (2004) [5] reported improved neurobehavioral function, increased frontal-occipital EEG coherence, and higher relative anisotropy in the left internal capsule following NIDCAP. Als et al. (2012) [6] reported improved neurobehavior, electrophysiological findings, and brain-structure measures among growth-restricted preterm infants receiving NIDCAP. McAnulty et al. (2012) [7] demonstrated more mature internal-capsule and cingulum fiber tracts at school age in infants who had received NIDCAP. McAnulty et al. (2013) [8] reported improved executive function, spectral coherence, and cerebellar volumes at school age following NIDCAP in growth-restricted preterm infants. Milgrom et al. (2010) [9] found that parent-sensitivity training was associated with lower apparent diffusion coefficient values and higher fractional anisotropy, suggesting more mature white-matter development. Because McAnulty et al. (2012) and McAnulty et al. (2013) represent follow-up analyses of related parent cohorts, these reports should be interpreted as complementary evidence rather than independent confirmation of intervention effects.

Single-Family Room Studies (Two Reports)

Pineda et al. (2014) [10] reported diminished hemispheric asymmetry, a trend toward lower EEG cerebral maturation scores at term-equivalent age, and lower language scores at two years among infants cared for in private rooms. Lester et al. (2014) [11] reported improved medical and neurobehavioral outcomes following transition to a single-family-room NICU; however, the study did not include structural MRI or DTI outcomes. Overall, evidence regarding single-family-room care was mixed, and direct structural neuroimaging evidence remained limited.

Sensory-Intervention Studies (Three Reports)

Lordier et al. (2019) [12] reported stronger resting-state functional connectivity within the salience, auditory, sensorimotor, thalamic, and precuneus networks following music intervention. de Almeida et al. (2020) [13] reported that music exposure was associated with maturation of white matter within the acoustic radiations, external capsule/claustrum/extreme capsule complex, and uncinate fasciculus, together with larger amygdala volumes. de Almeida et al. (2023) [14] demonstrated increased cortical fiber cross-section and greater orientation dispersion within paralimbic and auditory-association regions following music intervention. Although these studies consistently reported favorable neuroimaging findings, differences in imaging modality, study design, and outcome assessment limited direct comparison across studies.

Stress, Pain, and Prematurity-Comparison Studies (Four Reports)

Smith et al. (2011) [15] reported that greater NICU stress exposure was associated with reduced frontal and parietal brain width, altered diffusion measures, and altered temporal-lobe functional connectivity. Brummelte et al. (2012) [16] found that greater procedural pain exposure was associated with reduced white-matter fractional anisotropy and reduced subcortical gray-matter N-acetylaspartate/choline ratios. Ball et al. (2013) [17] compared preterm and term-born neonates and reported diminished thalamocortical connectivity following preterm birth but did not directly evaluate NICU stress exposure. Tortora et al. (2019) [18] demonstrated that early pain exposure was associated with reduced thalamic-somatosensory and insular-limbic functional connectivity. These observational studies demonstrate associations rather than causal effects, and residual confounding cannot be excluded.

Long-Term Follow-up Studies (Three Reports)

McAnulty et al. (2012) [7] demonstrated that neonatal NIDCAP was associated with neuropsychological, electrophysiological, and diffusion MRI differences that persisted to school age. McAnulty et al. (2013) [8] reported differences in executive function, EEG coherence, and cerebellar volume among school-age children who had received NIDCAP during the neonatal period. Thompson et al. (2014) [19] found that increased neonatal white-matter diffusivity in inferior occipital and cerebellar regions was associated with a greater risk of motor and executive-function impairment at seven years of age. These findings suggest that early neuroimaging measures may have prognostic relevance; however, follow-up attrition and limited sample sizes warrant cautious interpretation. A summary of the main MRI and DTI findings according to the intervention category is presented in Table 6.

**Table 6:** Summary of MRI/DTI outcomes by intervention category ↑ = increased/higher/more mature; ↓ = decreased/lower/less mature. Source: Prepared by the authors based on the included studies [5-19]. NIDCAP: Newborn Individualized Developmental Care and Assessment Program; DTI: diffusion tensor imaging

Category	Imaging/outcome findings
NIDCAP/developmental care	Several reports described favorable neurobehavioral, electrophysiological, diffusion, and selected structural neuroimaging findings following NIDCAP-based developmental care [5–9].
Single-family-room NICU	Mixed evidence: one study reported adverse cerebral maturation and language findings [10], whereas another reported improved medical and neurobehavioral outcomes without MRI/DTI findings [11].
Sensory interventions (music)	Music interventions were associated with stronger functional connectivity [12], improved auditory and emotional white-matter maturation and larger amygdala volume [13], and increased cortical microstructural complexity at term-equivalent age [14].
Stress and pain exposure	Observational studies reported that greater NICU stress or procedural pain exposure was associated with altered brain dimensions, diffusion measures, metabolites, and functional connectivity [15,16,18].
Prematurity comparison	Preterm birth was associated with diminished thalamocortical connectivity compared with term birth [17].
Long-term outcomes	Neonatal developmental care interventions and diffusion MRI findings were associated with neurodevelopmental outcomes at school age [7,8,19].

Meta-Analysis Feasibility

A quantitative meta-analysis was considered but was not feasible because of substantial methodological heterogeneityamong the included studies. Important differences existed in intervention type, neuroimaging modality, MRI acquisition protocols, regions of interest, outcome measures, follow-up duration, and reporting of variance estimates. In addition, some reports represented follow-up analyses of previously published parent cohorts, further limiting independent quantitative pooling. Therefore, findings were synthesized using a qualitative systematic review approach.

Discussion

This qualitative systematic review synthesized evidence from structural MRI, diffusion MRI, and selected fMRI studies evaluating the influence of enriched versus standard NICU environments on brain development in preterm infants. Across the included reports, NIDCAP-based developmental care, parent-sensitive developmental care, and structured music interventions were associated with favorable neurodevelopmental or neuroimaging findings in several studies, whereas evidence supporting single-family-room care remained inconsistent. Separate observational studies evaluating NICU stress and procedural pain demonstrated associations with less favorable neuroimaging findings; however, these findings should not be interpreted as establishing direct comparative or causal effects.

Studies evaluating NIDCAP-based developmental care consistently reported improvements in neurobehavioral function, diffusion MRI measures, electrophysiological findings, and selected structural neuroimaging outcomes. Follow-up reports suggested that some of these differences persisted into school age, indicating that early developmental care may have long-term neurodevelopmental relevance. However, because several publications represented follow-up analyses of previously published parent cohorts, these findings should be interpreted as complementary evidence rather than independent confirmation of intervention effectiveness.

Studies evaluating structured music interventions reported favorable findings involving functional connectivity, white-matter maturation, cortical microstructure, and selected regional brain volumes. Although these observations support the biological plausibility of sensory enrichment during early brain development, substantial variation existed in imaging modalities, intervention protocols, timing of assessment, and outcome measures. Consequently, direct comparison between individual studies remains limited.

Evidence regarding single-family-room NICU care was inconsistent. One study reported alterations in cerebral maturation and language outcomes, whereas another demonstrated improved medical and neurobehavioral outcomes without corresponding structural neuroimaging findings. These observations suggest that architectural redesign alone cannot be assumed to improve structural brain development and that individual components of developmental care should be evaluated independently.

Observational studies examining NICU stress and procedural pain exposure consistently reported associations with altered brain dimensions, diffusion abnormalities, metabolite changes, and disrupted functional connectivity. These findings highlight the potential importance of minimizing cumulative stress and painful procedures during neonatal intensive care. Nevertheless, because these studies were observational, residual confounding, differences in illness severity, treatment intensity, and other clinical factors cannot be excluded. Therefore, the observed associations should not be interpreted as evidence of direct causality.

Long-term follow-up studies demonstrated that neonatal neuroimaging findings and developmental-care interventions may remain associated with neurodevelopmental outcomes at school age. These observations support the potential prognostic value of neonatal neuroimaging while emphasizing the importance of continued long-term follow-up in future studies.

A quantitative meta-analysis was not performed because of substantial heterogeneity across the included reports. Important differences existed in intervention type, MRI acquisition parameters, diffusion techniques, functional imaging protocols, scanner field strengths, regions of interest, timing of imaging, follow-up duration, and reported outcome measures. The presence of follow-up publications arising from the same parent cohorts further limited the feasibility of quantitative pooling and independent effect estimation.

Overall, the available evidence suggests that individualized developmental care and structured music interventions maybe associated with favorable neurodevelopmental and neuroimaging outcomes in preterm infants. However, substantial methodological heterogeneity, relatively small sample sizes, follow-up publications from shared cohorts, and observational evidence for stress and pain exposures require cautious interpretation of the findings. Future multicenter prospective studies using standardized neuroimaging protocols, harmonized outcome measures, and adequately powered independent cohorts are needed before firm clinical recommendations can be established.

Strengths

This review followed PRISMA 2020 guidance and applied a predefined search strategy across two major databases. Intervention categories were clearly classified, and randomized and observational studies underwent structured risk-of-bias assessment using validated tools. The review also distinguishes intervention studies from observational stress- and pain-exposure studies, facilitating more transparent interpretation of the available evidence.

Limitations

Several limitations should be acknowledged. Considerable heterogeneity existed across the included studies with respect to intervention type, MRI acquisition protocols, diffusion imaging techniques, functional imaging methods, scanner field strengths, regions of interest, timing of neuroimaging, follow-up duration, and reported outcome measures. Some included reports represented follow-up analyses of previously published parent cohorts and therefore should not be interpreted as independent study populations. In addition, several observational studies evaluated associations between NICU stress or procedural pain and neuroimaging outcomes and remain susceptible to residual confounding. Differences in illness severity, clinical management, and sample size further limited direct comparison across studies. These methodological limitations are consistent with previous reports highlighting variability in neonatal neuroimaging acquisition, diffusion MRI quality, and outcome reporting across preterm imaging studies [23-27]. These limitations precluded quantitative meta-analysis and require cautious interpretation of the available evidence.

## Conclusions

This qualitative systematic review found that NIDCAP-based developmental care and structured music interventions were associated with favorable neurodevelopmental and neuroimaging findings in several included reports. Evidence regarding single-family-room care remained inconsistent, while separate observational studies associated greater NICU stress and procedural pain exposure with less favorable neuroimaging outcomes. Because of substantial heterogeneity, follow-up publications from some parent cohorts, and methodological limitations, these findings should be interpreted cautiously and should not be considered evidence of direct comparative or causal effects. Larger, well-designed prospective studies using standardized neuroimaging protocols are needed to strengthen the evidence base.
